# Land use change and forest management effects on soil carbon stocks in the Northeast U.S.

**DOI:** 10.1186/s13021-024-00251-7

**Published:** 2024-02-06

**Authors:** Lucas E. Nave, Kendall DeLyser, Grant M. Domke, Scott M. Holub, Maria K. Janowiak, Adrienne B. Keller, Matthew P. Peters, Kevin A. Solarik, Brian F. Walters, Christopher W. Swanston

**Affiliations:** 1 College of Forest Resources and Environmental Science, Michigan Technological University, Houghton, MI 49931 USA; 2 Northern Institute of Applied Climate Science, Houghton, MI 49931 USA; 3 American Forests, Washington, DC 20005 USA; 4 USDA Forest Service, Northern Research Station, St. Paul, MN 55108 USA; 5 Weyerhaeuser Company, Eugene, OR 97440 USA; 6 USDA Forest Service, Northern Research Station, Houghton, MI 49931 USA; 7 USDA Forest Service, Northern Research Station, Delaware, OH 43015 USA; 8 National Council for Air and Stream Improvement, Inc. (NCASI), Montréal, Québec H3A 3H3 Canada; 9 Office of Sustainability and Climate, USDA Forest Service, Washington, DC 20250 USA

**Keywords:** Deforestation, Reforestation, Forest management, Soil carbon, Northeast U.S., Forest harvest

## Abstract

**Background:**

In most regions and ecosystems, soils are the largest terrestrial carbon pool. Their potential vulnerability to climate and land use change, management, and other drivers, along with soils’ ability to mitigate climate change through carbon sequestration, makes them important to carbon balance and management. To date, most studies of soil carbon management have been based at either large or site-specific scales, resulting in either broad generalizations or narrow conclusions, respectively. Advancing the science and practice of soil carbon management requires scientific progress at intermediate scales. Here, we conducted the fifth in a series of ecoregional assessments of the effects of land use change and forest management on soil carbon stocks, this time addressing the Northeast U.S. We used synthesis approaches including (1) meta-analysis of published literature, (2) soil survey and (3) national forest inventory databases to examine overall effects and underlying drivers of deforestation, reforestation, and forest harvesting on soil carbon stocks. The three complementary data sources allowed us to quantify direction, magnitude, and uncertainty in trends.

**Results:**

Our meta-analysis findings revealed regionally consistent declines in soil carbon stocks due to deforestation, whether for agriculture or urban development. Conversely, reforestation led to significant increases in soil C stocks, with variation based on specific geographic factors. Forest harvesting showed no significant effect on soil carbon stocks, regardless of place-based or practice-specific factors. Observational soil survey and national forest inventory data generally supported meta-analytic harvest trends, and provided broader context by revealing the factors that act as baseline controls on soil carbon stocks in this ecoregion of carbon-dense soils. These factors include a range of soil physical, parent material, and topographic controls, with land use and climate factors also playing a role.

**Conclusions:**

Forest harvesting has limited potential to alter forest soil C stocks in either direction, in contrast to the significant changes driven by land use shifts. These findings underscore the importance of understanding soil C changes at intermediate scales, and the need for an all-lands approach to managing soil carbon for climate change mitigation in the Northeast U.S.

**Supplementary Information:**

The online version contains supplementary material available at 10.1186/s13021-024-00251-7.

## Background

Soil carbon (C) is an integral part of ecological, biogeochemical, and hydrologic processes within forest ecosystems, thus underpinning numerous ecological values and ecosystem services [1, 2]. In recognition of this importance and the critical role soils play in proposed climate change mitigation strategies, interest in the effects of land use change and forest management on soil C has increased ([3, 4].

High-level review papers on land use change, forest management, and soil C are numerous and have contributed to calls for climate change mitigation through C management (e.g., [5–11]). Quantitative reviews of afforestation, forest harvesting, fire management, and other practices have estimated broad-scale management effects upon soil C, and identified broad-scale factors responsible for variation in those effects [12–16]. However, reviews often provide generalizations that do not align with the findings of individual site-level studies. This disparity emphasizes the need for research on soil C management at intermediate (landscape to ecoregional) scales, which are often the focus of decision making by landowners, forest managers, and policymakers.

As new primary research continues to emerge, synthesis approaches are becoming increasingly useful for addressing soil C management at intermediate scales. The flexibility of synthesis tools is part of their utility; for example, meta-analysis enables estimating treatment effects across multiple studies while discerning factors that drive variation in these effects [17]. However, all meta-analyses are constrained by the available studies they synthesize,in the context of research on land use and C management, this means meta-analyses are helpful in identifying trends across select sites, but unable to address the diversity of conditions across intervening spaces [18]. Drawing in more extensive observational data, such as from soil survey and forest inventory programs, compensates for this problem. Compared to meta-analysis of published (relatively controlled) studies, observational survey or inventory datasets lack experimental control, may not possess desired auxiliary variables, and introduce other sources of variation that can obscure or confound trends. Nonetheless, observational data enable inferences over intervening areas that have not been reported in the literature, and auxiliary variables can be obtained from other sources to evaluate and contextualize meta-analysis results [19]. In pursuit of a more nuanced view of land use and C management, combining meta-analysis with observational data has been useful for downscaling from broad patterns (e.g., [14, 20] to the uniqueness of distinct ecoregions [21–24], and promises to find applications in still more.

**Fig. 1:**
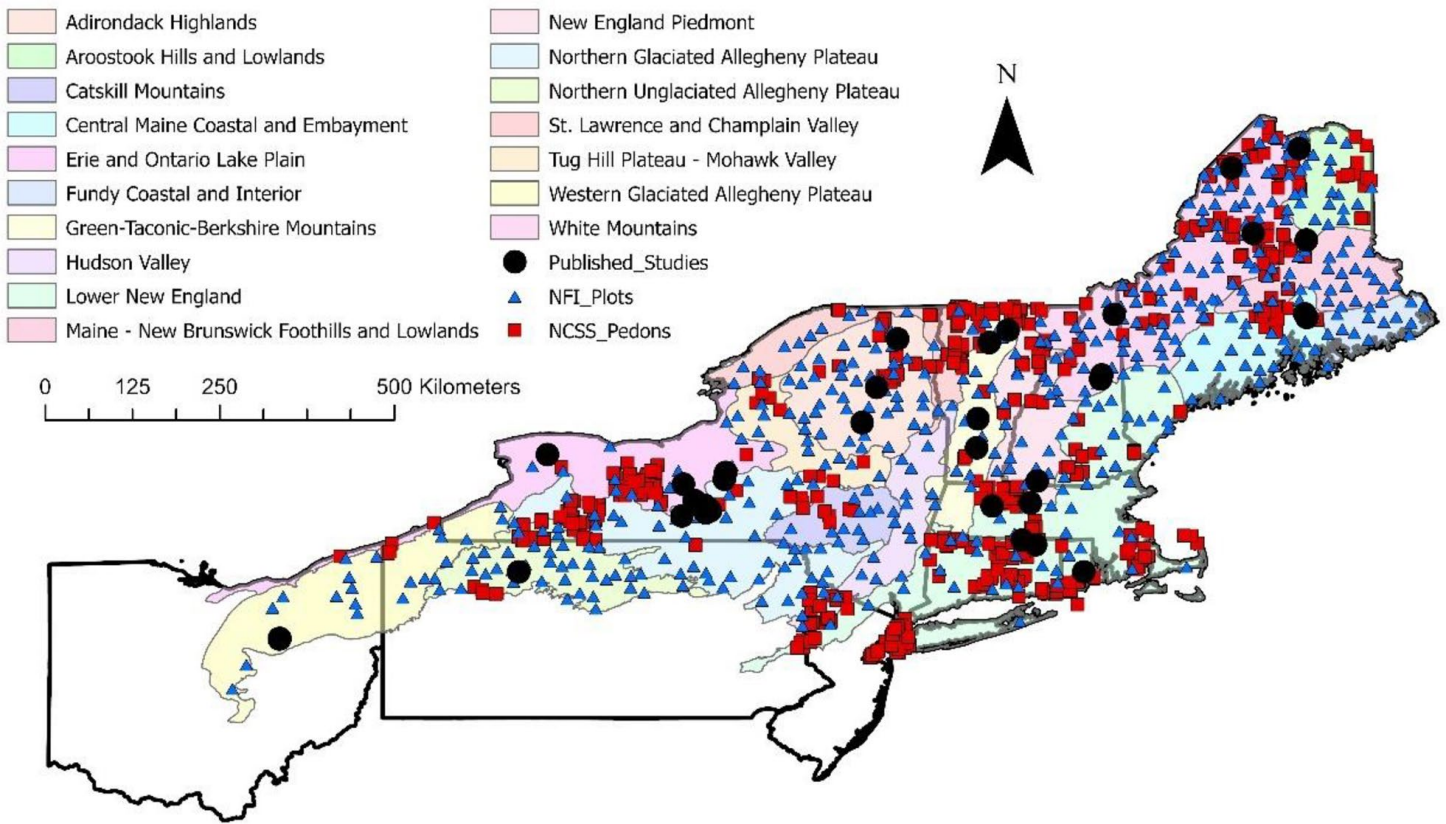
Map of the study area. Shaded polygons are ECOMAP Ecological Sections. Black points, which are approximate, represent the locations of study sites in papers reviewed for the meta-analysis. Red squares and blue triangles show locations of soil survey pedons and NFI plots, respectively

The U.S. Northeast (Fig. 1) is a 359,000 sq. km region that is home to nearly 50 million people (14% of the U.S. population), yet despite its large population, is ~ 75% forested (see “Study area" Sect. for description). From northern Pennsylvania and New Jersey to Maine, the forests of the Northeast are currently among the most C-dense in the U.S. [25]. But the large contemporary C stocks of these forest lands belie the region’s history. Native Americans managed the forests of the region for millennia before Euro-American colonists displaced them and largely deforested the region in the seventeenth–nineteenth centuries, resulting in large reductions in forest C stocks [26–28]. Agricultural abandonment and reforestation since that time have driven regional forest C stock increases, but these increases are slowing with time for two reasons: (1) forests are sequestering C more slowly as they mature, (2) deforestation for urban development and agriculture in the last several decades is once again causing forest area to decline across the Northeast [25, 29–31]. With these regional land use and C dynamics as the backdrop, scientific management, public perceptions, and policy discussions are more concerned than ever with questions of C balance and management in the Northeast U.S. Often, these considerations focus on forest management more than land use change, and on aboveground C rather than soils, which hold over half of total ecosystem C [25]. Such questions are also frequently discussed without considering broader land use change drivers and pressures that are critically important in a region where nearly 80% of forest lands are privately owned [32]. The present synthesis, representing the fifth in a series of ecoregional assessments, addresses soils—the dominant terrestrial C pool in the region—and examines the effects of both land use change and forest management.

## Results

### Meta-analysis: soil C stock change across land uses

**Fig. 2:**
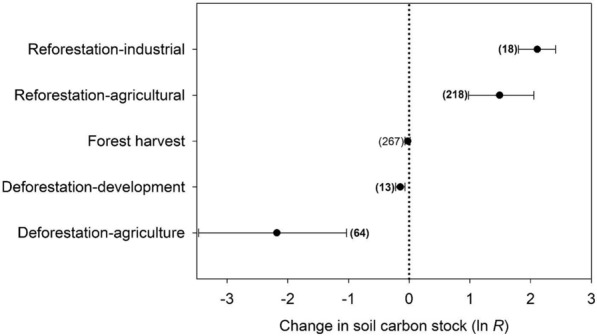
Meta-analytic main effects of land use and management treatments on soil C stocks, for all depths and horizons. The plot shows the mean effect size (as the ln-transformed response ratio *R*), bootstrapped confidence interval, and sample size for each treatment. Bold sample sizes indicate meta-analytic groups with confidence intervals not overlapping 0% change (indicated with a dotted vertical line)

The first of our three synthesis approaches, meta-analysis of published literature (see “Approach” and “Meta-analysis” Sects), indicated that the five land use and management treatments we were able to address with the available data had significantly different effects on soil C stocks (Fig. 2; *P* < 0.001; *Q*_*b*_ = 790; *Q*_*t*_ = 5791). Deforestation decreased soil C stocks, with (1) forest-to-agriculture driving significantly larger losses than (2) forest-to-developed land use change. Conversely, reforestation increased soil C stocks, with similar effect sizes for reforestation of (3) formerly agricultural vs. (4) mining industry lands. Forest harvesting (5) had no statistically significant effect (*P* > 0.05) on soil C stocks.

**Fig. 3:**
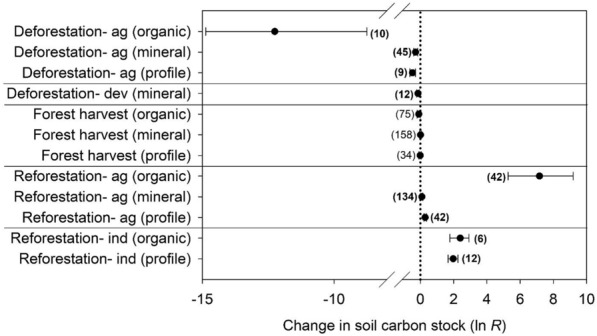
Meta-analytic effects of land use and management treatments on soil C stocks, shown separately for organic horizons, mineral soils, and whole soil profiles. The plot shows the mean effect size (as the ln-transformed response ratio *R*), bootstrapped confidence interval, and sample size for each treatment and depth with sufficient data. Bold sample sizes indicate meta-analytic groups with confidence intervals not overlapping 0% change (indicated with a dotted vertical line)

Meta-analysis indicated that land uses also differed in the depth distribution of their effects on soil C stocks (Fig. 3). Deforestation for agriculture caused significant declines in soil C stocks in all analyzed portions of the profile (see “Meta-analysis” Sect), with significantly larger losses from O horizons than mineral soil horizons or whole soil profiles. Reforestation following agriculture showed the opposite trend, with very large positive effects on O horizons, modest but still statistically significant increases for mineral soil horizons, and intermediate values for soil profiles. Reforestation formerly mining industry lands caused large soil C stock increases in O horizons and soil profiles (no mineral soil horizons were reported), while the significant, negative effects of deforestation for development were based entirely on results published for mineral soil horizons. Forest harvesting did not significantly affect soil C stocks in O horizons, mineral soil horizons (either collectively or by horizon), or soil profiles.

### Meta-analysis: a closer look at agricultural reforestation

**Fig. 4:**
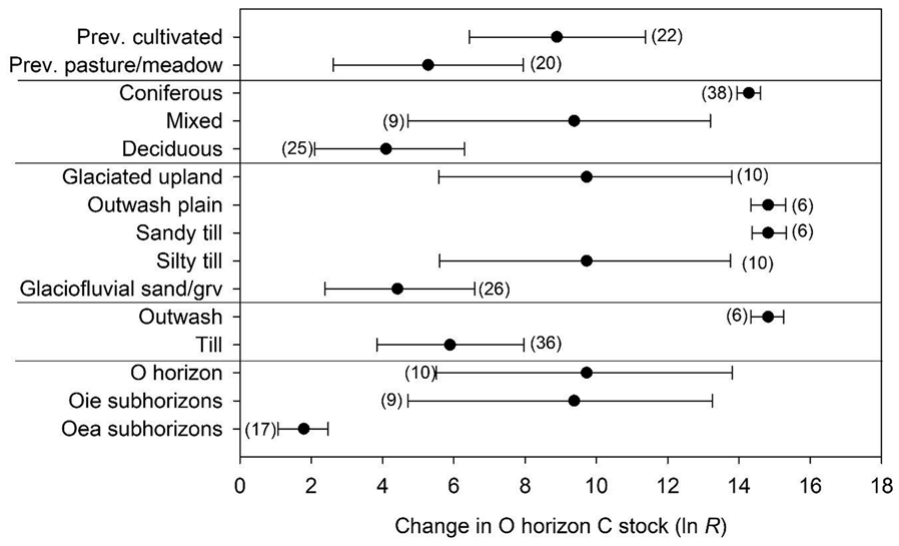
Meta-analytic effects of post-agricultural reforestation on organic horizon C stocks, as dependent on several predictor variables. The plot shows the mean effect size (as the ln-transformed response ratio *R*), bootstrapped confidence interval, and sample size for each group. Groups plotted are a subset of those showing statistically significant differences in effect sizes

Reforestation following agriculture was one of two land uses with enough data to support more detailed meta-analyses. Organic horizon C stock increases during reforestation varied according to most of the potential predictor variables analyzed. Table 1 presents these statistically significant results, and indicates the large amounts of total (*Q*_*t*_) and between-group (*Q*_*b*_) heterogeneity resulting from near-zero agricultural O horizon C stock values (see “Meta-analysis” Sect. for information about heterogeneity and response ratio calculations). A subset of the factors that were statistically significant predictors of this variation are illustrated in Fig. 4. Previously cultivated soils gained significantly more O horizon C than soils previously managed as pasture or meadow; O horizon gains were largest for reforestation by coniferous forests, intermediate for mixed forests, and least for deciduous forests. Ecological Province, Section, and Subsection were significant predictors of variation in O horizon C gains; these ecoregional patterns were associated with statistically significant meta-regression *P* values for mean annual temperature (larger O horizon gains in warmer areas), mean annual precipitation and elevation (significantly smaller gains in wetter areas and at higher elevations). Organic horizon C gains also were significantly larger in flatter vs. steeper, and S-facing vs. E-facing topographic settings (which were the only two slope aspects that could be explicitly tested). Landform and parent material influences were evident as larger O horizon gains for soils formed in sandy outwash materials (vs. finer-textured soils formed in till and glaciated uplands). Carbon increases in O horizons overall were driven by increases in more litter-like, less decomposed materials (Oie subhorizons) more than increases in older, more decomposed organic materials (Oea subhorizons). Lastly, statistically significant effects of soil taxonomy (Suborder and Great Group) indicated that O horizon C gains during reforestation were influenced by a range of integrative, soil-specific factors.

**Table 1:** Meta-analysis results for potential predictors of C stock changes during post-agricultural reforestation

Organic horizons				Mineral soils (all horizons)			
Factor	*Qb (Qm)*	*Qt*	*P*	Factor	*Qb (Qm)*	*Qt*	*P*
Study identity	953.7	1800	< 0.001	Study identity	4.5	18.9	0.72
Previous land use	136.7	1800	< 0.001	Previous land use	0.1	18.9	0.71
Stand-level forest type group	684.4	1800	< 0.001	Stand-level forest type group	0.5	18.9	0.78
Stand-level forest type	885.6	1800	< 0.001	Stand-level forest type	2.2	18.9	0.83
Ecoprovince	847.3	1800	< 0.001	Ecoprovince	1.1	18.9	0.77
Ecosection	847.3	1800	< 0.001	Ecosection	1.2	18.9	0.88
Ecosubsection	953.7	1800	< 0.001	Ecosubsection	1.8	18.9	0.94
Mean annual temperature	586.6	1800	< 0.001	Mean annual temperature	0.0	18.9	1.00
Mean annual precipitation	90.3	1800	< 0.001	Mean annual precipitation	0.0	18.9	0.95
Elevation	465.4	1800	< 0.001	Elevation	0.0	18.9	0.87
Slope steepness group	556.4	1800	< 0.001	Slope steepness group	0.4	18.9	0.83
Aspect class	516.8	1800	< 0.001	Aspect class	1.0	18.8	0.60
Landform group	614.0	1800	< 0.001	Landform group	0.8	18.9	0.94
Parent material	410.1	1800	< 0.001	Parent material	0.2	18.9	0.90
Surface geology	614.0	1800	< 0.001	Surface geology	0.9	18.8	0.82
Soil Order	827.2	1800	< 0.001	Soil Order	1.0	18.6	0.60
Soil Suborder	856.5	1800	< 0.001	Soil Suborder	1.4	18.3	0.85
Soil Great Group	953.7	1800	< 0.001	Soil Great Group	2.9	16.7	0.71
Soil Subgroup	953.7	1800	< 0.001	Soil Subgroup	2.9	16.7	0.71
Organic subhorizon	200.1	1800	< 0.001	Soil master horizon	1.6	18.9	0.80
				Soil texture	2.9	18.8	0.89
Wetness group	827.2	1800	< 0.001	Wetness group	2.0	18.9	0.57
Drainage index	510.1	1800	< 0.001	Drainage index	0.9	18.9	0.36
Productivity index	465.8	1800	< 0.001	Productivity index	0.1	18.9	0.80

Statistics include the between-group (*Q*_*b*_) or continuous model (*Q*_*m*_) heterogeneity, total heterogeneity (*Q*_*t*_), and* P* value for each predictor variable

Among studies of post-agricultural reforestation, O horizons and mineral soil horizons were similar in that no predictor variable exceeded the explanatory power (as *Q*_*b*_/*Q*_*t*_) of study identity as a predictor of C stock changes during reforestation. On the other hand, mineral soil horizons were very different from O horizons in that none of their predictors of variation tested had statistically significant *P* values. In the case of some predictor variables, certain categorical groups showed mineral soil C stock changes with confidence intervals that did not overlap 0% (Additional file 1: Table S1), and the nuances of interpreting statistical significance of meta-analysis results are discussed in Methods Sect. “Meta-analysis”.

### Meta-analysis: a closer look at forest harvesting

Among studies of forest harvesting—the other treatment with sufficient data for more detailed meta-analyses—none of the predictor variables tested significantly affected soil C stocks (Table 2). In the case of both organic and mineral soil horizons, some predictor variables revealed individual categorical groups with effect sizes and confidence intervals that did not overlap 0% change in soil C stocks (Additional file 1: Table S2). For soil bulk density (Db), neither organic nor mineral soil horizons showed statistically significant effects of harvesting, and none of the variation among mineral soils was significantly related to any of the tested predictor variables (O horizons were too few to analyze). Some individual categorical groups had Db response ratios with effect sizes and confidence intervals that did not overlap 0% change (Additional file 1: Table S3).

**Table 2:** Meta-analysis results for potential predictors of C stock changes in response to forest harvesting, for organic vs. mineral soil horizons

Organic horizons				Mineral soils (all horizons)			
Factor	*Qb(Qm)*	*Qt*	*P*	Factor	*Qb(Qm)*	*Qt*	*P*
Study identity	7.5	16.5	0.58	Study identity	2.0	16.8	0.98
Time since harvest	0.9	16.7	0.83	Time since harvest	1.3	16.9	0.73
Harvest system	0.0	16.7	0.94	Harvest system	0.0	16.9	0.92
Basal area removal fraction	1.0	16.7	0.62	Basal area removal fraction	0.0	16.9	0.99
Harvest type	1.7	16.7	0.64	Harvest type	0.1	16.9	0.99
Materials removed	0.0	16.7	0.98	Materials removed	0.2	16.9	0.89
Season of harvest	0.3	16.5	0.88	Season of harvest	0.4	16.9	0.81
Felling methods	0.1	16.7	0.78	Felling methods	0.2	16.9	0.64
Hauling methods	1.2	16.7	0.75	Hauling methods	0.3	16.9	0.96
Traction type	0.3	16.7	0.88	Traction type	0.6	16.9	0.76
Landscape-level forest type	0.2	16.7	0.64	Landscape-level forest type	0.4	16.8	0.54
Stand-level forest type group	1.0	16.7	0.59	Stand-level forest type group	0.4	16.9	0.82
Stand-level forest type	5.4	16.7	0.25	Stand-level forest type	1.0	16.9	0.90
Mean annual temperature	1.3	16.7	0.26	Mean annual temperature	0.9	16.9	0.33
Mean annual precipitation	0.0	16.7	0.97	Mean annual precipitation	0.2	16.9	0.66
Ecoprovince	1.2	16.7	0.54	Ecoprovince	0.5	16.8	0.78
Ecosection	4.1	16.7	0.66	Ecosection	1.3	16.8	0.93
Ecosubsection	5.7	16.5	0.89	Ecosubsection	1.5	16.7	1.00
Elevation	2.2	16.7	0.14	Elevation	0.1	16.9	0.74
Slope steepness group	4.6	16.7	0.20	Slope steepness group	0.1	16.9	0.99
Aspect class	3.5	16.7	0.32	Aspect class	0.1	16.9	0.99
Landform group	5.5	16.7	0.48	Landform group	1.6	16.9	0.90
Parent material	5.5	16.5	0.48	Parent material	1.4	16.9	0.92
Surface geology	3.2	16.7	0.67	Surface geology	0.2	16.8	1.00
Soil Order	0.8	16.7	0.86	Soil Order	0.7	16.9	0.88
Soil Suborder	0.9	16.7	0.93	Soil Suborder	0.7	16.9	0.95
Soil Great Group	3.2	16.5	0.79	Soil Great Group	0.9	16.9	0.97
Soil Subgroup	9.1	16.4	0.70	Soil Subgroup	2.4	16.9	1.00
Organic subhorizon	3.0	16.7	0.70	Soil master horizon	1.0	16.9	0.91
				Soil texture	0.9	16.9	0.99
Wetness group	0.3	16.7	0.85	Wetness group	0.5	16.9	0.80
Drainage index	1.4	15.5	0.28	Drainage index	0.0	16.6	0.87
Productivity index	1.9	15.5	0.17	Productivity index	0.1	16.6	0.75

Statistics include the between-group (*Q*_*b*_) or continuous model (*Q*_*m*_) heterogeneity, total heterogeneity (*Q*_*t*_), and* P* value for each predictor variable

### Observational data: broader context and evaluation of meta-analytic trends

The first of our two observational datasets, consisting of soil survey pedons harmonized with GIS data, supported parametric statistical analyses indicating a range of factors that influence soil C stocks across the study area (Table 3). To aid in comparing to meta-analysis data, we focused on two distinct portions of the profile: surface horizons (O and A horizons), and whole soil profiles. In general, variation in soil C stocks was more explainable in surface horizons (O and A horizons, n = 793) than in whole soil profiles (to refusal or 50 cm depth, n = 873). However, multiple regression models for both portions of the profile had adjusted *R*^2^ values indicating that over half the variance remained unexplained (see Sects. “Synthesis of soil pedon and GIS data” and “Statistical analysis of NCSS and NFI data” for analytical details). The optimal surface horizon model indicated that C stocks were larger in organic soils (Histosols) and where surface horizons consisted of organic soil materials; where surface soils were mineral (i.e., A horizons), categorical texture class variables indicated larger C stocks for finer-textured soils and smaller C stocks for coarser soils. Several variables related to land use, management, and vegetation were present in the surface horizon model: compared to reference forest lands, soil C stocks on pasture/hay lands and recently deforested lands were smaller, soil C stocks in recently harvested forests were larger, and soil C stocks decreased with increasing aboveground biomass. Compared to the default parent material dummy variable (coarse till), several parent materials were associated with smaller surface horizon C stocks, including alluvial, colluvial, and residual deposits and fine-textured till. The same soil C associations with parent material were evident for whole soil profiles, which additionally indicated that outwash (by definition coarse) and proglacial deposits (whether coarse or fine) were associated with smaller soil C stocks. Surface horizon and soil profile models both showed that C stocks increased with elevation; profile C also exhibited a positive relationship with mean annual precipitation. Regarding land use, profile C was less on barren lands and developed open spaces than in reference forests (which were the dummy variable default), and profile C increased with aboveground biomass. The remaining variables in the optimal whole soil profile C model were related to soil taxonomy, with Mollisols, Alfisols, and Inceptisols holding significantly less C than Spodosols (the dummy variable default) and Histosols holding more.

**Table 3:**
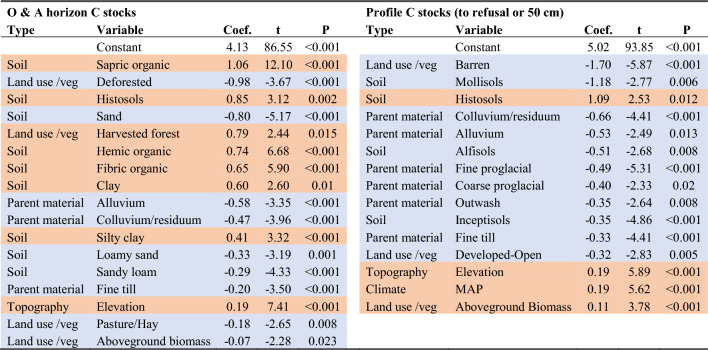
Variables included in optimal best subsets regression models of O&A horizon (*R*^2^ = 0.45) and profile total (*R*^2^ = 0.32) C stocks (*P* = 0.001 for each), based on soil pedon and GIS data

Variables are sorted by their relative strength of influence (in terms of standardized slope coefficients) and color-coded to distinguish those that are positively (orange) vs. negatively (blue) related to C stocks. See Additional file 1: Table S4 for the complete list of variables included in the selection pool for this modeling procedure

The second of our two observational datasets (from the U.S. Department of Agriculture Forest Service [USDA FS] National Forest Inventory [NFI] program) revealed two key results supporting meta-analytic harvest trends, and one which, like soil pedon data, ran counter to them. First, NFI data indicated that neither of the sampled mineral soil depth increments (0–10 and 10–20 cm) had significantly different C stocks as a function of past harvesting (t-test, *P* = 0.82 and *P* = 0.90, respectively; see “National Forest Inventory dataset” and “Statistical analysis of NCSS and NFI data" Sections), which was generally congruent with meta-analysis results indicating no significant harvest effects on C stocks in mineral soil horizons. Second, NFI data indicated a similar relationship between stand age and O horizon C stocks as detected by meta-analysis (Fig. 4), with forests aged 26–50 years having significantly lower C stocks than older stands. This result followed the meta-analytic pattern that forests harvested 26–50 years prior to sampling (roughly the latter 2–3 decades of the twentieth century for this dataset) had a negative O horizon C stock response ratio with confidence intervals that did not overlap 0% change (Fig. 4, Table 4). In terms of the key difference, NFI data (like the soil survey pedon + GIS data) indicated that O horizons in harvested forests held significantly more C (t-test, *P* < 0.001, 21 vs. 14 Mg C ha^−1^) than forests that had not been recently harvested.

**Table 4:** Summary of study conclusions

Finding	Confidence	Management, C accounting, & policy considerations
1. Deforestation decreases soil C stocks	H	Soil C losses are largest at the surface of the profile and diminish with depth; declining regional forest cover in recent decades suggest land use change is having increasingly negative effects on the regional forest sector C budget
2. Reforestation increases soil C stocks	H	Soil C gains are largest at the surface of the profile and diminish with depth; across the region, large areas of forest that were once farmed are likely on a trajectory of long-term soil C increase
3. Soil C increases with reforestation vary by site	H	Landform, soil, and vegetation influence O horizon C gains; site-level uncertainty in rates is tied to unknown factors (potentially including past agricultural practices)
4. Forest harvesting does not affect soil C stocks	H	Harvesting in general does not affect soil C; outcomes to the contrary are highly exceptional and not predictable based on natural factors or silvicultural practices
5. O horizon C may be vulnerable to harvest in select settings	M	For Spodosols, northern & boreal tree communities, steep slopes, and S-facing aspects, there is some (highly limited) evidence for O horizon C losses; low-risk-tolerance management may minimize interventions in such settings while increasing them in settings with no evidence for O horizon C losses
6. Existing management guidelines benefit soil C	M	Soil- and water-protecting practices that are already implemented most of the time as best management practices or for legal compliance are not explicitly targeting soil C protection, but are likely promoting it
7. Partial harvest systems require further research	H	Clearcutting is studied far more frequently than it occurs; more research on more extensive partial harvests is needed to better constrain soil C outcomes
8. Soil C is one aspect of forest climate vulnerability	M	Silviculture and soil C management can facilitate forest climate adaptation and mitigation by placing soils, topography, and tree communities in landscape context to define goals and objectives for diverse stands and settings

Confidence ratings indicate high (H) versus medium (M) confidence in strength of inference, based upon the degree of support across data sources

## Discussion

### Land use change, forest harvesting, and soil C change in a regional context

This analysis focused on the Northeast is the most data-rich, intensive effort in our series of ecoregional investigations of land use and forest management effects on soil C stocks in the U.S. [21–24]. Among these studies, the Northeast is also unique as the only ecoregion where there is no detectable effect of forest management on soil C stocks, either overall or as a function of place-based or practice-specific factors. In addition to the absence of any forest harvesting effects, another key finding of the present analysis is the clear evidence of the effects of land use change on soil C stocks. Where deforestation has occurred (whether for agriculture or urban development), it has caused significant soil C losses, and these losses are likely substantial at a regional scale given the increasing urbanization and declines in forest cover in New England in recent decades [29, 33]. Contrary to the negative effect of deforestation, our analysis demonstrates that reforestation consistently increases soil C stocks in the Northeast, and reveals place-based factors that explain variation in those increases. These findings enhance the results of detailed site-level studies in the region (e.g., [34, 35] by revealing broader relationships between soil C stock changes and those place-based factors, which may be useful in prioritizing reforestation efforts. For example, planting conifers on outwash soils drives larger O horizon C gains than hardwood regrowth on finer-textured till soils, and because outwash soils tend to be less agriculturally productive, they may be more likely to support such a land use change. This place-based variation aside, long-term soil C increases are probably widespread in the Northeast because so much of the region was farmed decades to centuries in the past. Reforesting soils thus represent a component of the regional forest C sink that is rarely quantified as research tends to focus on more readily measured aboveground C pools [27, 36, 37]. The exclusion of soils from forest C stock assessments in the Northeast is unfortunate in light of the large proportion of total ecosystem C that soils hold, and the more rapid rates of soil C gain after reforestation than other ecoregions of the U.S. [20, 25]. Collectively, these findings have two important implications for future land use change and C management in the Northeast. First, from a wider perspective, urbanization of farmland is also probably C-negative, because it drives agriculture to become more fossil fuel intensive and to expand at the expense of forestland [38, 39]. Second—and considering forestland specifically—where climate change driven declines in forest health or other environmental changes may contribute to deforestation, forest management can help mitigate these threats to the maintenance of forestland area and C stocks. Ultimately, maintaining forest land as forest, whether or not it is managed, is a way to maintain the largest terrestrial C stock in the region.

### Northeast forest soils and global change

Forest soils in the Northeast have larger C stocks than in most other areas of the U.S. [40], with profile totals ranging from 60 to 241 Mg C ha^−1^ in our meta-analysis dataset. Thesesoils frequently include well-developed O horizons, averaging 12% of the whole-profile C stock, and high C concentrations in mineral soils, both of which may be due to some combination of acid parent material, productive vegetation, and generally cool, wet climate [41–45]. As large C stocks often translate to high variability, and in any case, hinder the detection of small treatment effects [46], the large baseline soil C stocks of Northeast forest soils may partly explain why they appear resistant to forest harvest. However, several of these C-positive factors—notably, the large proportion of C held in unprotected surface organic matter and the role of historical climate and vegetation inputs—imply several mechanisms whereby climate change could alter soil C stocks in the long term. First, warming and wetting accelerate microbial activity, lengthen decomposition seasons, and increase hydrologic exports of C in runoff, surface and groundwater [47, 48]. Second, the potential for increased fire activity due to the increasingly episodic nature of precipitation may make O horizons vulnerable, particularly in coniferous forests with thicker litter layers, forests that are overstocked or have high fuels density, or otherwise unhealthy forests [49–52]. Third, climate change impacts on forests and their C are occurring in synergy with other global change drivers. For example, as Northeast forest soils recover from prior decades of elevated nitrogen (N) and acid deposition, which increased soil C via biogeochemical mechanisms, climate change may interact with changing nutrient demand, microbial community composition and biochemistry to destabilize SOM [53, 54]. Although not yet widely observed, one mechanism for these interactions is the potential for coupled vegetation-microbial “mining” of N-rich SOM due to simultaneous increases in primary production and N limitation [55–57]. Climate change is also increasing nonnative earthworm populations and associated invasive species in the Northeast, significantly altering soil C stocks and their vertical distribution [58, 59]. In the context of these synergistic global change drivers, regional reductions in soil C stocks, particularly in O horizons, appear likely. Thus forest managers and other resource professionals will increasingly have to contend with soils that are losing organic matter for reasons beyond their direct control, even as their own forest management decisions have little direct effect on soil C [60].

### Forest climate vulnerability, soil C management, and adaptation

The potential for climate change to affect soil C through a variety of direct, biogeochemical, disturbance, and land use change mechanisms points to several ways that management can be used to mitigate soil C vulnerability, while also mitigating overall forest vulnerability to climate change [61, 62]. Reiterating that meta-analysis revealed no statistically significant effects of harvesting on soil C stocks, we draw attention to several of its more nuanced findings that may inform management options. For example, O horizon C stocks for (1) Spodosols and (2) forest types with climate-vulnerable northern tree species had slightly negative effect sizes, with confidence intervals that did not overlap 0% change (Additional file 1: Table S2). This implies that Spodosols supporting northern and boreal tree species may have some level of soil C vulnerability to harvesting, and may argue for alternative management approaches for these climate-vulnerable soil and forest types. One such approach could be to move away from harvesting on Spodosols and instead manage for climate-vulnerable northern forest types more on Inceptisols, which have smaller C stocks (Table 3) and showed no harvest effect on their O horizons. Other options may be to harvest northern forest types on Spodosols only under certain settings (e.g., N-facing slopes) or meteorological conditions (e.g., frozen ground). In either case, placing soils, topography, tree species, and silvicultural details in landscape context can facilitate management to jointly mitigate soil C and climate vulnerabilities.

Moving beyond merely mitigating vulnerabilities, soil C management can also be used to increase adaptive capacity in the face of climate change. For example, active forest management to favor diverse mixtures of southern species on inherently more productive soils, such as Alfisols, targets soils that have less C to begin with (Table 3) and are not affected by harvest, while facilitating transition to more climate-adapted forest types [63–65]. Where factors such as topography or drainage favor more cold-adapted soil and forest types, careful harvest removals can increase structural and compositional complexity—traits which increase adaptive capacity—while maintaining these climate-vulnerable systems [66–68].

Setting aside climate change, our meta-analysis revealed several additional patterns that can inform forest management in the heterogeneous landscapes of the Northeast. These more nuanced patterns should be interpreted cautiously as they are based upon meta-analytic groups with nonzero, negative response ratios rather than predictor variables with *P* < 0.05 statistical significance (Additional file 1: Tables S2, S3, Sect. “Meta-analysis"). The first of these patterns were tendencies for harvesting (1) large proportions of stand basal area or (2) on steeply sloping terrain or (3) on south-facing slopes to diminish O horizon C stocks (Additional file 1: Table S2). In light of these potential vulnerabilities, increasing retention in stands on steep terrain, especially on south-facing slopes, may minimize the risk of soil C losses. The second pattern of note among these more nuanced findings was that light to moderate basal area reductions (vs. heavy basal area reductions) were associated with a tendency for mineral soil Db to increase (Additional file 1: Table S3). This may be because harvesting fewer trees results in fewer harvest residues (e.g., tree tops, limbs) being available to protect harvesting and hauling trails from vehicle traffic [69]. In this hypothetical scenario, reducing harvest intensity (or forgoing harvest altogether) on steep slopes can be used to minimize risks to soil C stocks and physical properties, while furthering other objectives such as those related to water quality or wildlife habitat. At the same time, an adequate supply of wood can be maintained by increasing removals in flatter settings, at no apparent risk to soil C or physical properties, as our meta-analysis revealed no changes for any other harvest intensity or slope steepness groups. Overall, these patterns support harvest prescriptions that are usually already employed to support soil and water quality guidelines [70, 71], indicating that even when these guidelines are implemented for other reasons, they are compatible with if not beneficial to soil C management.

### Caveats

In each ecoregional soil C assessment that has comprised this series of analyses, we have used multiple independent approaches to assess confidence in results by judging their consistency across data sources and methods. Here, we discuss and discount a trend that emerged in two of the three data sources in the present analysis, which we believe to be an artifact that highlights the challenge of using observational data to understand forest C change and its drivers. That is, while meta-analysis revealed no statistically significant harvest effects, the two observational datasets indicated that O horizon C stocks were significantly larger in forests harvested in the last 2–3 decades than those without recent management. Most of all, we discount this trend because meta-analysis is a more robust technique, being based upon individual published studies that have been designed to minimize the influence of the many sources of variation in soil C stocks, thus maximizing their ability to detect any effects of harvest. Observational survey and inventory programs are decidedly not designed to provide such a statistical framework, much less one that can rigorously address questions as specific as we focus on here. Relying on observational data to assess change in a case such as this thus risks a Type 1 error or “false positive,” especially given the strongly unbalanced design of our observational datasets: forests harvested in the past 2–3 decades represented only 3% and 12% of the observations in the soil pedon/GIS and NFI datasets, respectively. With such small sample sizes, it is reasonably likely that observations of harvested soils are directionally skewed by factors that influence soil C stocks in their own right. Such factors may have an even larger influence on our estimates of soil C change if they are confounded with geographic or site factors that make a forest more likely to be harvested. The small sample sizes of recently harvested sites in our two observational datasets also highlight another important point about the results of our analysis. The estimated regional area of harvested forests based on our observational datasets (3–12%) compares well with a geospatial analysis of silvicultural practices that placed total harvested area in the range of 2–17%, across states, over approximately the same period [72]. In the same analysis, clearcutting represented 21% of harvested area regionally, whereas 12 of 20 published papers that we found for inclusion in our meta-analysis (60%) reported the results of this most intensive type of forest harvesting. In combination, the lack of any overall meta-analytic harvest effects, the preponderance of less intensive types of harvesting, and the modest extent of harvesting in general, indicate that harvesting has little potential to affect soil C in the Northeast.

Another caveat relates to soil sampling depths. As described in Sect. “Meta-analysis”, published studies presented us with data for a wide range of sampling approaches and depths. Our prior ecoregional soil C assessments have indicated significantly different management effects on different soil horizons, with O horizons typically being most responsive [21–24]. Thus, in the present analysis, we used the same approach to systematically categorize data across sampling approaches into a set of depth-related variables for further investigation. Surprisingly, soil horizons did not differ significantly in their responses to harvest (Table 2). The reasons for this lack of a significant effect are as uncertain as the reasons for the lack of an overall harvest effect, which has at least been implied in certain settings or for certain practices in other ecoregions. The mutually consistent O horizon increase in our two observational datasets, one of which (the soil survey pedon dataset) samples by horizon and the other of which (the national forest inventory dataset) samples by a horizon / depth hybrid approach, may imply that there may be functional interactions between soil horizons. However, as discussed in the prior paragraph, we discount these results due to the potential for spatial design problems, which should be expected in a region where a large share of the harvesting is done in northern coniferous forest types with large O horizon C stocks [42, 44, 73–75].

### Insights from regional case studies

While our synthesis demonstrates regional consistency in the lack of harvest impacts on soil C stocks, detailed site-level case studies sometimes arrive at other conclusions, which inform our results and their implications. The Hubbard Brook Experimental Forest Watershed 5 clearcut study is one of these studies, and it has been the focus of several mechanistic and long-term resampling studies. Data for our meta-analysis come from the most recent resampling (1998) of the experiment, which was implemented in 1983 [76]. In this longitudinal study, O horizon and mineral soil C stocks have both changed significantly, albeit over different timescales. During the initial years after treatment, O horizons declined due to mixing with the surface mineral soil [77]. Organic horizons have recovered since that time, yet mineral soil C stocks have declined sufficiently to drive net losses in whole profile soil C [76]. Molecular characterization has revealed that loss of mineral soil C has been accompanied by replacement of more degraded (likely slower-cycling) SOM by more recent, residue-derived C inputs [78], a process supported by SOM fractionation results from a clearcut experiment at the nearby Bartlett Experimental Forest [79]. While these results together provide a pattern and a mechanism for situations in which soil C stocks decline after harvesting, they are tempered by the wider results of our analysis, which suggests that they are exceptional. This may be a result of the experimental treatment in the Watershed 5 study, which represents intensive harvesting (whole-tree harvest of all stems down to 5 cm diameter) that is almost never seen in operational forestry in the region [72].

**Fig. 5:**
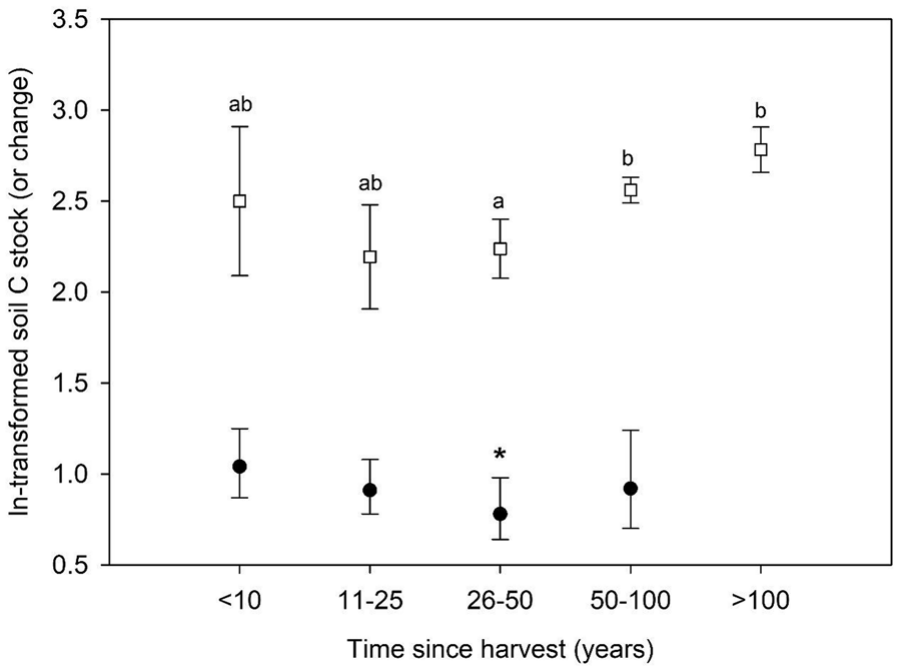
Organic horizon C stocks as a function of time since harvest based on meta-analysis results (filled circles) and NFI plots (open squares). Time since harvest (years) is presented as a categorical variable (age class group), due to the difficulty of precisely constraining years since harvest among the published studies used for meta-analysis. Y-axis values for NFI plot data are ln-transformed C stocks and 95% confidence intervals; for meta-analysis results, y-axis values are C stock changes (ln-transformed response ratios and bootstrapped confidence intervals). Letters denote statistical significance of pairwise comparisons (Tukey test) for NFI plot data; for meta-analysis results, the asterisk indicates the age class group with confidence intervals not overlapping 0% change (*R* = 1.00)

Like Hubbard Brook and Bartlett, Yanai et al. [80] studied clearcut-origin northern hardwoods on Spodosols in northern New England, in an effort to critically assess chronosequence results and constrain temporal trajectories of organic matter stocks following harvest. In their study, the authors justifiably sampled combined O and A horizons as part of what they defined as the “forest floor,” for reasons including mixing of organic and mineral horizons during harvesting. When resampled after 15 years, individual stands showed every possible trajectory of change, including increases, decreases, and no change in forest floor organic matter. This result highlights a frequent criticism of the chronosequence approach. One of the important conclusions of the study remains the argument that stands harvested at different points in time differ not only in the circumstances of their individual harvests, but in the environmental conditions in which they have developed since. As rates of climate and other global change drivers continue to increase, this argues for a conservative approach to interpreting soil C stock data from forests differing in their ages. With this in mind, our results showing that O horizon C stocks vary with stand age (Fig. 5) provide limited basis for predicting C stock changes into the future for harvested forests, much the same as aboveground C should also be interpreted with caution when extrapolating past conditions into the future [81]. Rather, it is more appropriate to view chronosequences as current snapshots of C stocks in forests with different histories, and from there, to allow those current conditions to guide future management. In this regard it is clear that the oldest forests are the most effective C reserves at present, due to their large above- and belowground C stocks. Younger forests meet a range of other objectives in landscape-scale management, with their more rapid rates of C accumulation in this region and elsewhere [82] providing opportunities to transfer C from rapidly aggrading aboveground biomass to more slowly cycling soil pools. Our analysis indicates that regionally, harvesting older forests to make them better suited to increased rates of climate change and disturbance does not come at the expense of C stored in the soil.

Across the 7 states in the region analyzed by Belair et al. [72], 72–100% of the area harvested in the past 2–3 decades has consisted of various types of partial harvesting, making primary studies of these other silvicultural interventions very important. Increasingly, the soil C literature is turning in this direction, with studies addressing more prevalent treatments such as single- and group-tree selection, crop tree release, various types of thinning, and shelterwood harvests. Two recent partial harvest studies, both in northern hardwoods, provide several nuanced insights that help advance the clearcutting-biased literature on this topic [83, 84]. First, neither of these studies detected significant changes in total organic horizon C stocks (though Puhlick and Fernandez [83] reported fine-fraction O horizon declines). Second, both studies identified inputs of C, via harvest residues, as a possible mechanism for sustaining O horizon C stocks during the period following harvesting. Third, both studies suggested that microclimatic changes after harvesting could lead to altered soil C in the future, especially with increased climate change, and Puhlick and Fernandez [83] specifically referenced using harvest residues to physically protect soils from climate-intensified precipitation events. Importantly, those authors identified a trade-off between broadcasting harvest residues to mitigate weather and microclimate effects vs. concentrating them on trails to minimize machine traffic impacts. Our finding that light to moderate harvests tended to increase mineral soil Db, while heavy basal area reductions had no effect (Additional file 1: Table S3) highlights this tradeoff: partial harvests generate fewer residues, requiring managers to make difficult choices among competing uses. Ultimately, the outcomes of these difficult choices for soil C will be most favorable when the same choice is not made in all situations, much in the same way that management is most effective when planned and implemented at a landscape scale. At such a scale, prescriptions for individual stands will naturally differ based on site conditions, with a balanced range of management actions across the landscape mosaic meeting multiple objectives.

## Conclusions

The key inferences of this study are summarized in Table 4. Across the landscape mosaics of the Northeast U.S., soils are losing C where forests are being converted to other land uses and gaining it where forests are gradually recovering from historic deforestation. In the case of deforestation, conversion from forest to agriculture causes larger soil C stock declines per unit area than does deforestation for urban development. Soil C stock increases under reforestation are driven by a combination of known and unknown site-specific factors, including physiography, forest type, and past agricultural management practices. In general, forest harvesting has no effect on soil C stocks, and no place-based or practice-specific factors reveal any significant or systematic deviation from this overall trend, though a small minority of individual studies indicate significant changes in soil C stocks. Overall, interactions between individual site histories (e.g., disturbance, management) and place-based factors (e.g., physiography, soil type) mediate the effects of land use and forest management on soil C stocks. Recognizing these influences and using them to prioritize management strategies and actions at landscape levels (with examples provided herein) can promote C management as a tool in climate change adaptation and mitigation in this region.

## Methods

### Study area

Following previously published assessments [21–24], we delineated the study area for this analysis using the USDA FS ECOMAP system [85, 86]. Our focus was on glaciated northeastern U.S. states with regionally consistent climate, physiography, and vegetation, as constrained by the boundaries of several previously published ecoregional assessments [1, 23]. We synthesized data from within the 19 ECOMAP Ecological Sections comprising the study area, which entirely encompasses the states of Maine (ME), New Hampshire (NH), Vermont (VT), Massachusetts (MA), Rhode Island (RI), Connecticut (CT), and New York (NY), and includes portions of New Jersey (NJ), Pennsylvania (PA), and Ohio (OH, Fig. 1). Ecological Sections tier beneath the Province level in the hierarchical ECOMAP system and are summarized below.

The climate of the study area ranges from warm to hot continental, with cold winters and warm summers. Mean annual air temperatures range from 3 to 11º and mean annual precipitation from 850 to 1400 mm. Climatic extremes occur in interior mountain areas, which are generally colder and wetter, and climatic moderation occurs near the maritime influence of the Atlantic Ocean. Approximately three-fourths of the region is forested, with diverse cover types ranging from boreal conifers in the north and at high elevations, to mesic conifer-northern hardwoods mixtures across the majority of the study area and at intermediate elevations, to oak (*Quercus* spp.)-hickory (*Carya* spp.) and oak-pine (*Pinus* spp.) cover types in the southernmost and coastal ecological sections. Elevations range from sea level to 1913 m above sea level. The geology of the study area consists of a wide range of sedimentary, igneous, and metamorphic rock types occurring as mountains, ridges, valleys, cuestas, monadnocks, plateaus, and peneplains. Across most of the study area, these bedrock features are buried beneath glacial drift deposited during Wisconsinan glaciation *ca.* 28,000–14,000 years before present. Postglacial lakes and their outbursts, as well as erosion and alluvial deposition during the Holocene have modified the landscape in many areas. The most extensive soil orders of the region are Spodosols and Inceptisols, which are generally more common in areas that are cooler, wetter, and more topographically rugged; Alfisols and sporadic Mollisols are found in flatter, more clay-rich settings such as lakeplains and major river valleys. Histosols are found in interior and coastal wetlands, Entisols are found in wetlands and on modern floodplains, and Ultisols are present to a limited extent, especially in residual and colluvial parent materials near the southern limit of continental glaciation. Further and more detailed description of the study area’s climate, vegetation, geology, and soils is available in McNab et al. [86].

### Approach

We used synthesis methods detailed in previous ecoregional papers [21–24] and refined modestly for the current analysis. Methods included: (1) effect size meta-analysis of data from published papers; (2) synthesis of soil survey pedon observations with GIS information; (3) analysis of NFI data from plots in which soils, biomass, and other ecosystem properties were measured. We employed these methods as follows. First, we used meta-analysis to quantify the effects of five distinct land uses on soil C stocks: deforestation for agriculture, deforestation for urban development, reforestation after agriculture, reforestation on former industrial lands, and forest harvest. Second, using meta-analysis, we examined these effects in greater detail, quantifying their magnitude and uncertainty for three a priori defined portions of the soil profile: organic horizons, mineral soils, and whole soil profiles. Subsequently, for the two land uses with sufficient data (agricultural reforestation, forest harvest), we tested a range of potential predictor variables (Table 1 and electronic supporting information) for their ability to explain variation in soil C stock changes. Finally, following all meta-analyses, we analyzed soil pedon/GIS and NFI data as two independent sources of information, in order to assess the level of support for trends indicated by meta-analysis, and place meta-analysis results in the context of baseline soil C stocks and their controls across the study area.

### Meta-analysis

We synthesized data from 35 relevant papers, published between 1997 and 2023, identified through an extensive literature review (see Additional files1 and 2; Fig. 1). As with our prior meta-analyses, we followed a predetermined protocol for assessing each publication found during our literature review to determine its suitability. To be included, each paper had to: (1) provide control and treatment values for soil C (concentration or stock) or bulk density, for at least one relevant treatment; (2) offer adequate metadata to constrain locations and use as potential predictor variables; (3) present response data not included in previous studies; (4) fall within the study area. In this analysis, we introduced two new features compared to previous assessments. First, we incorporated bulk density (Db) as a specific response parameter of interest. Second, in several cases, we directly contacted study authors to obtain data not reported in, but underpinning several forest harvest papers from the region [73, 83, 84, 87, 88]. These personal communications were necessary to bring summarized data from these important regional papers to the same level of site-specific detail found in the majority of other forest harvest papers (or their associated Additional file 2: Datasets).

From all 35 papers, we extracted control and treatment soil C (or Db) values and utilized them to calculate effect sizes (as the *ln*-transformed response ratio *R*). As in our other published meta-analyses, these response ratios span a wide range of forest types, soil depths, amount of time elapsed since experimental treatments, and other sources of variation, both known and unknown. Response ratios also vary in terms of how their control conditions were defined. Reflecting the dominant land use history of the region, nearly all comparisons were between recently harvested forests vs. 60–100 year old second-growth forests; the remainder were a handful of studies that compared recently harvested forests to forests with no known Euro-American disturbance or management history. To analyze the data, we used MetaWin 3 [89] to conduct fixed-effects, categorical and continuous meta-analyses [17, 90], with bootstrapped confidence intervals determined using the Student’s t distribution [91, 92]. We chose to conduct an unweighted meta-analysis, which maximizes data availability, as weighted meta-analyses require sample size and variance statistics reported ~ 50% of the time in this literature. Furthermore, we did not assume that the data met parametric assumptions of a weighted meta-analysis.

In this meta-analysis, soil organic carbon (SOC) stock (Mg C ha^−1^) was our response variable of specific interest. When data were not reported in those units we converted them, as needed, using the same basic approaches as our other assessments [21–24], refined for the current region. For studies reporting soil organic materials as the concentration of soil organic matter (%SOM, derived from loss on ignition; 22 of 614 total response ratios), we converted %SOM to %SOC using soil order-specific factors and models from Boyle et al. [93]. For papers that reported soil C as %SOC, we derived a relationship between %SOC and Db for the 243 observations that reported both, and used the resulting model to predict Db from %SOC for papers only reporting the latter (%SOC,41 of 614 total response ratios). Based on the diagnostic shape of the %SOC vs. Db relationship, and principles outlined in Federer [94], we used an exponential decay model to fit the data. The resulting model (1) had *r*^2^ = 0.90, *P* < 0.001, and a standard error of the estimate of 0.13, for soils with C concentrations ranging from 0.17 to 48.77% and Db values ranging from 0.02 to 1.78 g cm^−3^.

1 $${\text{Db}}\, = \,0.0{867}\, + \,{1}.{1178 }*{\text{ exp}}\left( { - 0.{1266 }* \, \% {\text{SOC}}} \right)$$

We then computed SOC stock (Mg C ha^−1^) as the product of the reported %SOC, the predicted Db, and the reported sampling interval depth or horizon thickness. For studies reporting soil organic materials as a thickness measurement (3 response ratios, all organic horizons), we obtained the average organic horizon C concentration for the reported soil series from Soil Series Data Explorer (https://casoilresource.lawr.ucdavis.edu/sde/), used it to predict Db, and then computed SOC stock as the product of the predicted %SOC and Db values and the reported organic horizon thickness. In three studies (k = 33 response ratios) involving land use change, either the control or treatment value for organic horizon C stock was 0; we set these values equal to 0.00001 Mg C ha^−1^ in order to be able to compute a response ratio for these observations.

To evaluate place-based and practice-specific factors that may influence treatment effects on soil C stocks, we extracted a wide range of ecological, geographic, climatic, experimental, and methodological predictor variables from each paper. When needed, we looked up missing study site information in other publications from the same sites, or using geocoordinates reported in the papers, or by accessing information about the soil series reported from those study sites, via the USDA Natural Resources Conservation Service (USDA NRCS) online Official Soil Series Descriptions (https://soilseries.sc.egov.usda.gov/osdname.aspx). When key information was missing, but geocoordinates were provided, we used GIS lookups (Sect. “Synthesis of soil pedon and GIS data”) to obtain the necessary attributes for each study. The meta-analysis database we developed is provided as electronic supporting information.

Our strategy for categorizing soil sampling depths requires detailed description, because we tested for effects of depth in the soil profile in two ways (i.e., using two different variables). First, recognizing a fundamental difference between organic soil horizons, mineral soil horizons, and soil profiles, we categorized each response ratio according to a high-level “soil material” variable (organic, mineral, or profile). For the second approach, we first recorded either the genetic horizon (e.g., Oe, Oa, A, Bw1) or sampling increment (as depth range in cm) for each response ratio. Then, we placed genetic horizons and sampling depths into a “soil master horizon” variable. Probable master horizons included the master horizons O, A, E, B, C, as well as their various combinations (e.g., EB, BC). For papers reporting data by horizon, categorizing response ratios to their master horizon was straightforward; for papers reporting data by depth increment, we correlated each reported depth increment to its probable master horizon, based upon associated methods descriptions or soil series descriptions. When soil C stocks were reported for increments greater than 50 cm total depth, we summed them and categorized them as profiles, which we distinguished between mineral profiles (mineral soils only) and whole profiles (mineral soils plus organic horizons).

As in prior published assessments, we used meta-analysis to identify significant predictors of variation in soil C stock responses to management, which is completed statistically by parsing variation into within-group (*Q*_w_) and between-group heterogeneity (*Q*_b_) and inspecting corresponding *P* values [95]. Grouping variables that have large *Q*_b_ relative to *Q*_w_ are statistically significant (*P* < 0.05) and explain a larger share of total variation among all studies (*Q*_t_). However, we recognize that the statistical significance of *P* values is only one way to assess significance of meta-analysis results. In this study, in addition to identifying statistically significant predictors variation based on their *P* values, we were also concerned with identifying individual groups of response ratios that did not have predictors with statistically significant *P* values, but which were significantly different from zero percent change based on their bootstrapped confidence intervals not overlapping zero. In all meta-analyses, we imposed a small sample size criterion, electing a priori to exclude from discussions of statistical or practical significance any group that showed statistical significance (or was significantly different from zero percent change) but had fewer than k = 5 response ratios.

### Synthesis of soil pedon and GIS data

We complemented the experimental strength of meta-analysis, which generates strong inferences for a relatively limited number of intensively studied sites, with an extensive soil pedon dataset for our study area. These were data for geo-located soil pedons from the USDA NRCS, National Cooperative Soil Survey (NCSS) Database (July 2019 version), including latitude, longitude, soil taxonomy, and physical and chemical properties of individual soil horizons according to Schoeneberger et al. [96] and Burt et al. [97]. Data from the NCSS Database span decades of soil survey,as in prior papers [20–24], we only used pedons from 1989-present to increase concurrence with other datasets used in our analyses. In addition to the data contained in the NCSS Database, we used GIS to extract the following attributes for each geo-located NCSS pedon: land cover from the most closely coincident version of the National Land Cover Dataset [98–100], aboveground biomass density from the National Biomass Carbon Dataset (NBCD2000; [101, 102]), mean annual temperature (MAT) and precipitation (MAP) for the 1981–2010 period [103], landscape-level forest type [104], surface geology [105], integrated soil moisture [106], drainage [107] and productivity [65] indices, and elevation, slope aspect, and slope gradient from a 30 m digital elevation model (US Geological Survey 2023 [108]). To more precisely constrain recent land use and management history than is possible using the 250-m resolution, 5-year interval NLCD products, we used a combination of the USDA FS Landscape Change Monitoring System [109] and Google Earth Pro. This entailed using the LCMS to flag all pedon geolocations that experienced a “Fast Loss” in forest cover during the period of record (1985–2021) and then manually inspecting all available aerial and satellite imagery (1984–2019) for those pedon locations in Google Earth Pro to attribute specific types of forest change (i.e., partial harvest, heavy harvest, or deforestation for development).

As in prior published assessments, we used gap-filling techniques to estimate Db for soil horizons in the NCSS Database that did not possess measured values. Using an initial dataset of n = 4542 individual horizons from 1282 NCSS pedons, we developed a Db—%C relationship from the n = 1405 horizons with measured Db and %C values for the fine earth fraction (soil passing a 2 mm sieve). For Db, we preferentially used db_od (oven-dry mass divided by oven-dry volume) and, in limited cases, used oven-dry mass divided by field-moist volume (db_fmstw) where only that variant was available. For %C, we preferentially used the following: (1) %organic C measured directly, or (2) %total C minus %inorganic C, or (3) assumed %total C = %organic C, for samples with no measured %inorganic C but pH < 7.0. We excluded the very small number of soils with pH > 7.0 and no reported value for %inorganic C, as in these cases it would have been impossible to calculate %organic C. Next, we derived an exponential decay model following the same approach as described in Sect. “Statistical analysis of NCSS and NFI data”, generating a resulting Db—%C relationship, Eq. (2), that had *r*^2^ = 0.52, *P* < 0.001, and a standard error of the estimate of 0.21, for soils with %C ranging from 0.00 to 62.43% and Db ranging from 0.19 to 2.44 g cm^−3^.

2 $${\text{Db }} = \, 0.{9}0{8}0 \, + \, 0.{8}00{2 }*{\text{ exp}}\left( { - 0.{2752 }* \, \% {\text{SOC}}} \right)$$

For all horizons, we then computed SOC stock (Mg C ha^−1^) as the product of %C, Db, and horizon thickness. For tests involving whole profile C stocks, we summed the C stocks of individual horizons to a depth of 50 cm or refusal (e.g., by bedrock), assuming that C was homogenously vertically distributed in the case of horizons that spanning 50 cm and thus mathematically truncating these horizons at a depth of 50 cm in the profile C stock summation.

### National forest inventory dataset

We complemented our meta-analysis and NCSS pedon + GIS datasets with observational data from the USDA FS NFI. The NFI plots are the basis for what is widely known as the Forest Inventory and Analysis (FIA) program, which provides data from an equal-probability sample of all forest lands in the conterminous U.S. Across the conterminous U.S, there is one permanent plot on approximately every 2400 ha, randomly placed within a systematic hexagonal sampling frame [110]. All NFI plots with at least one forest condition are measured every 5–7 years in the eastern U.S. Soils are sampled from a subset of these plots, according to a protocol in which organic horizons are sampled first and mineral soils are then sampled in depth increments of 0–10 and 10–20 cm. The NFI plot design ensures no systematic bias with regard to location, ownership, composition, soil, physiographic factors, or other characteristics. We obtained data for this analysis from a July 2022 query of the FIA Database for records of organic horizon, 0–10 cm and 10–20 cm mineral soil C stocks (all in Mg C ha^−1^). To ensure data consistency, we constrained the query to plots that were at least 75% under the same condition, excluding plots divided along sharp boundaries with different stand age, slope, wetness, etc., such that local variation in such factors would misrepresent conditions at the actual location of soil sampling. Moreover, we only used the most recent observation available in the FIA Database [111] of each long-term NFI plot, and only plots observed since 2000, aligning the NFI data with meta-analysis and NCSS pedon datasets. Similar to the NCSS pedon data, we used GIS lookups of NFI plot locations to obtain the following attributes, from the same sources: mean annual temperature and precipitation [103], surface geology [105], and soil taxonomic order [112]. Altogether, our NFI datasets for this ecoregion included n = 470 organic horizons and n = 319 mineral soils (Phase 3 or P3 plots).

### Statistical analysis of NCSS and NFI data

To complement the non-parametric meta-analysis of experimental data from published papers, we used parametric statistics (SigmaPlot 14, SYSTAT Software, San Jose, CA US) to analyze observational NCSS and NFI data. To identify factors influencing baseline SOC stocks in (1) O and A horizons and (2) whole soil profiles (to 50 cm or refusal), we analyzed NCSS data using best subsets regressions to identify variables with statistically significant categorical or continuous relationships with SOC stocks. In these analyses, we coded categorical predictors as dummy variables and standardized continuous predictors by subtracting the mean and dividing by the standard deviation. Before beginning model selection, we defined the optimal model as having the highest adjusted *R*^2^ and being comprised entirely of variables with significant partial *P* values. We set these criteria in order to identify the largest possible suite of factors influencing SOC stocks, while protecting against over-fitting by including variables that increased total proportion of variance explained, but themselves lacked significant relationships with SOC stocks. For NFI data, we used t-tests to compare control vs. harvested C stocks for the three soil depths reported by the FIA program, and ANOVA with the Tukey post-hoc test to compare mean O horizon C stocks across forest age class groups. For all of these statistical analyses, we used *ln-*transformations as necessary to normalize response variables, and in all analyses, we set *P* < 0.05 as the threshold for statistically significant results.

### Supplementary Information

**Additional file 1: Table S1. **Factors associated with non-zero changes in mineral soil C stocks during post-agricultural reforestation. For each factor, levels appearing under the Gain column indicate groups of response ratios with a positive effect size and a bootstrapped confidence interval that did not overlap 0% change; levels under the Loss column indicate groups of response ratios with a negative effect size and a bootstrapped confidence interval that did not overlap 0% change. See section “Meta-analysis” for interpretation of these marginally significant results. **Table S2. **Factors associated with non-zero changes in soil C stocks with harvest. Results are shown separately for O horizons (upper half of the table) vs. mineral soils (lower half of the table). For each factor, levels appearing under the Loss column indicate groups of response ratios with a negative effect size and a bootstrapped confidence interval that did not overlap 0% change; levels under the Gain column indicate groups of response ratios with a positive effect size and a bootstrapped confidence interval that did not overlap 0% change. See section “Meta-analysis” for interpretation of these marginally significant tendencies. **Table S3. **Factors associated with non-zero mineral soil Db changes with harvest. For each factor, levels appearing under the Decline column indicate groups of response ratios with a negative effect size (decrease in Db) and a bootstrapped confidence interval that did not overlap 0% change; levels under the Increase column indicate groups of response ratios with a positive effect size (increase in Db) and a bootstrapped confidence interval that did not overlap 0% change. See section “Meta-analysis” for interpretation of these marginally significant tendencies. **Table S4. **Selection pool of variables available for inclusion in best subsets regression models of C stocks in O&A horizons (left half of table) vs. whole soil profiles (right half of table) in the soil survey pedon + GIS dataset. For categorical factors, which were coded as dummy variables, parentheses indicate the levels of each factor and the dummy variable default is indicated with bold text; all other factors are continuous.

**Additional file 2. **compiled meta-analysis dataset used to test for land use change and harvest effects on soil carbon stocks.

## Data Availability

Two of the three data sources used in this analysis (NFI plot data and NCSS pedon data) are publicly available. The third, being the meta-analysis dataset synthesized from peer-reviewed literature, is included as additional files information.
