# Supplementary material for: Land use change and forest management effects on soil carbon stocks in the Northeast U.S
Source: Carbon Balance Manag. 2024 Feb 6;19:5. doi: 10.1186/s13021-024-00251-7 (PMC10845599; doi:10.1186/s13021-024-00251-7)
Supplement: Supplementary file 1 — Additional file 1: Table S1. Factors associated with non-zero changes in mineral soil C stocks during post-agricultural reforestation. For each factor, levels appearing under the Gain column indicate groups of response ratios with a positive effect size and a bootstrapped confidence interval that did not overlap 0% change; levels under the Loss column indicate groups of response ratios with a negative effect size and a bootstrapped confidence interval that did not overlap 0% change. See section “Meta-analysis” for interpretation of these marginally significant results. Table S2. Factors associated with non-zero changes in soil C stocks with harvest. Results are shown separately for O horizons (upper half of the table) vs. mineral soils (lower half of the table). For each factor, levels appearing under the Loss column indicate groups of response ratios with a negative effect size and a bootstrapped confidence interval that did not overlap 0% change; levels under the Gain column indicate groups of response ratios with a positive effect size and a bootstrapped confidence interval that did not overlap 0% change. See section “Meta-analysis” for interpretation of these marginally significant tendencies. Table S3. Factors associated with non-zero mineral soil Db changes with harvest. For each factor, levels appearing under the Decline column indicate groups of response ratios with a negative effect size (decrease in Db) and a bootstrapped confidence interval that did not overlap 0% change; levels under the Increase column indicate groups of response ratios with a positive effect size (increase in Db) and a bootstrapped confidence interval that did not overlap 0% change. See section “Meta-analysis” for interpretation of these marginally significant tendencies. Table S4. Selection pool of variables available for inclusion in best subsets regression models of C stocks in O&A horizons (left half of table) vs. whole soil profiles (right half of table) in the soil survey pedon + [file 13021_2024_251_MOESM1_ESM.docx]

**Supplementary Information**

**Table S1.** Factors associated with non-zero changes in mineral soil C stocks during post-agricultural reforestation. For each factor, levels appearing under the Gain column indicate groups of response ratios with a positive effect size and a bootstrapped confidence interval that did not overlap 0% change; levels under the Loss column indicate groups of response ratios with a negative effect size and a bootstrapped confidence interval that did not overlap 0% change. See section 5.3 for interpretation of these marginally significant results.

| **Factor** | **Gain (significantly >0% change)** | **Loss (significantly <0% change)** |
| --- | --- | --- |
| Study identity | Clark & Johnson 2011; Norris 2012; Barnett et al. 2020 | Pacaldo et al. 2013; Wang et al. 2016 |
| Previous land use | cultivated |  |
| Stand-level forest type | maple-beech-birch |  |
| Ecoprovince | M211- Adirondack-NE-Mixed-Coniferous Forest/Alpine Meadow |  |
| Ecosection | M211C- Green, Taconic, Berkshire Mountains |  |
| Ecosubsection | 221Ib- Erie-Ontario Lake Plain; M211Cc- Berkshire-Vermont Upland |  |
| Landform | outwash plain |  |
| Surface geology | glaciofluvial sand/gravel |  |
| Slope class | nearly level |  |
| Slope aspect | S-facing slopes | W-facing slopes |
| Parent material | till |  |
| Soil Subgroup | Typic Dystrudepts | Glossic Hapludalfs |
| Soil horizon | A horizon; B horizon |  |
| Soil texture | fine-loamy; sandy |  |

**Table S2.** Factors associated with non-zero changes in soil C stocks with harvest. Results are shown separately for O horizons (upper half of the table) vs. mineral soils (lower half of the table). For each factor, levels appearing under the Loss column indicate groups of response ratios with a negative effect size and a bootstrapped confidence interval that did not overlap 0% change; levels under the Gain column indicate groups of response ratios with a positive effect size and a bootstrapped confidence interval that did not overlap 0% change. See section 5.3 for interpretation of these marginally significant tendencies.

| **O horizons** | **Loss (significantly <0% change)** | **Gain (significantly >0% change)** |
| --- | --- | --- |
| Study identity | Yanai et al 2000; Hoover 2011; Hamburg et al. 2019; Puhlick et al. 2023 | Warren & Ashton 2014 |
| Time since harvest | 26-50 years |  |
| Basal area removal fraction | high proportion of basal area removed |  |
| Harvest type | clearcut or thinning | shelterwood |
| Felling methods | use of processor for felling |  |
| Landscape-level forest type | aspen/fir/cedar/birch |  |
| Stand-level for. type grp. | deciduous forest |  |
| Stand-level forest type | maple/beech/birch |  |
| Ecoprovince | M211-Adirondack-New England Mixed Forest Coniferous Forest-Alpine Meadow |  |
| Ecosection | M211A- White Mountains |  |
| Ecosubsection | M211Ac- Maine Central Mountains; M211Ad- White Mountains | 221Ag- Southeast New England Coastal Hills and Plains |
| Topography | steep slopes |  |
| Topography | S-facing slopes |  |
| Landform | glaciated upland |  |
| Parent material | sandy till | melt-out till |
| Soil Order | Spodosols |  |
| Soil Suborder | Orthods |  |
| Soil Great Group | Haplorthods |  |
| Soil Subgroup | Typic Haplorthods |  |
| Soil horizon | Oe, Oa horizons |  |
| **Mineral soils** | **Loss (significantly <0% change)** | **Gain (significantly >0% change)** |
| Study identity |  | Yanai et al. 2000 |
| Time since harvest |  | 26-50 years |
| Harvest removals | logs-only harvest |  |
| Stand-level forest type |  | maple-beech-birch |
| Ecoprovince | 221- Eastern Broadleaf Forest |  |
| Ecosection | 221A- Lower New England | M211A- White Mountains |
| Ecosubsection | 221Ag- SE New England Coastal | M211Af- Connecticut Lakes |
| Topography | S-facing slopes |  |
| Soil Order | Inceptisols |  |
| Soil Suborder | Udepts |  |
| Soil Great Group | Dystrudepts |  |
| Soil Subgroup | Typic Dystrudepts | Typic Haplorthods |
| Soil horizon | E horizons |  |

**Table S3.** Factors associated with non-zero mineral soil Db changes with harvest. For each factor, levels appearing under the Decline column indicate groups of response ratios with a negative effect size (decrease in Db) and a bootstrapped confidence interval that did not overlap 0% change; levels under the Increase column indicate groups of response ratios with a positive effect size (increase in Db) and a bootstrapped confidence interval that did not overlap 0% change. See section 5.3 for interpretation of these marginally significant tendencies.

| **Variable** | **Decline (significantly <0% change)** | **Increase (significantly >0% change)** |
| --- | --- | --- |
| Study identity |  | Bustos & Egan 2011; Puhlick & Fernandez 2020; Richmond et al. 2022 |
| Harvest system |  | uneven-aged management |
| Basal area removal fraction |  | light to moderate removals |
| Harvest type |  | selection or thinning |
| Felling methods |  | use of processor for felling |
| Hauling methods |  | use of both skidder & forwarder |
| Stand-level for. type grp. |  | northern hardwood-conifer; spruce-fir |
| Time since harvest | 50-100 years | 0-10 years |
| Ecoprovince |  | 211-Northeastern Mixed Forest |
| Ecosection |  | 211F- Northern Glaciated Allegheny Plateau |
| Ecosubsection | M211Af- Connecticut Lakes | 211Fb- Central Allegheny Plateau  M211Aa- International Boundary Plateau  M211Ab- St. John Upland |
| Topography | nearly level; very steep | moderately to strongly sloping |
| Topography |  | S-facing slopes |
| Landform |  | glaciated upland |
| Parent material |  | till |
| Soil Great Group |  | Fragiudepts |
| Soil texture |  | loamy to coarse-loamy |
| Soil horizon | E horizon | A horizon; EB horizon |

**Table S4.** Selection pool of variables available for inclusion in best subsets regression models of C stocks in O&A horizons (left half of table) vs. whole soil profiles (right half of table) in the soil survey pedon + GIS dataset. For categorical factors, which were coded as dummy variables, parentheses indicate the levels of each factor and the dummy variable default is indicated with bold text; all other factors are continuous.

| **O & A horizons** |  |  | **Soil profiles** |  |
| --- | --- | --- | --- | --- |
| **Group** | **Variables** |  | **Group** | **Variables** |
| Soil | pH; texture (fibric hemic, sapric, sand, loamy sand, sandy loam, **loam**, silt loam, silt, silty clay loam, silty clay, clay); order (Entisols, Inceptisols, **Spodosols**, Alfisols, Ultisols, Mollisols, Histosols); drainage index; productivity index; integrated moisture index |  | Soil | Order (Entisols, Inceptisols, **Spodosols**, Alfisols, Ultisols, Mollisols, Histosols); drainage index; productivity index; integrated moisture index |
| Parent material | (**Coarse till**, fine till, coarse proglacial, fine proglacial, outwash, alluvium, colluvium/residuum, coastal/eolian) |  | Parent material | (**Coarse till**, fine till, coarse proglacial, fine proglacial, outwash, alluvium, colluvium/residuum, coastal/eolian) |
| Land use / veg | Land use (barren, cultivated, pasture/hay, wetland, previously cultivated wetland, deforested, ag to developed, developed, developed-open, **forest**, harvested forest, shrub, grassland); aboveground biomass |  | Land use / veg | Land use (barren, cultivated, pasture/hay, wetland, previously cultivated wetland, deforested, ag to developed, developed, developed-open, **forest**, harvested forest, shrub, grassland); aboveground biomass |
| Topography | Elevation; slope gradient; slope aspect (**N**, E, S, W) |  | Topography | Elevation; slope gradient; slope aspect (**N**, E, S, W) |
| Climate | Mean annual temperature; mean annual precipitation |  | Climate | Mean annual temperature; mean annual precipitation |

**Meta-analysis references**

Barnett SE, Youngblut ND, Buckley DH. Soil characteristics and land-use drive bacterial community assembly patterns. Fems Microbiology Ecology. 2020;96(1).

Bustos O, Egan A. A Comparison of Soil Compaction Associated with Four Ground-Based Harvesting Systems. Northern Journal of Applied Forestry. 2011;28(4):194-8.

Carpenter R, Ward EB, Wikle J, Duguid MC, Bradford MA, Ashton MS. Soil nutrient recovery after shelterwood timber harvesting in a temperate oak hardwood forest: Insights using a twenty-five-year chronosequence. Forest Ecology and Management. 2021;499.

Clark JD, Johnson AH. Carbon and Nitrogen Accumulation in Post-Agricultural Forest Soils of Western New England. Soil Science Society of America Journal. 2011;75(4):1530-42.

Delgado BLM, Kenefic LS, Weiskittel AR, Fernandez IJ, Benjamin JG, Dibble AC. Northern mixedwood composition and productivity 50 years after whole-tree and stem-only harvesting with and without post-harvest prescribed burning. Forest Ecology and Management. 2019;441:155-66.

Fuss CB, Lovett GM, Goodale CL, Ollinger SV, Lang AK, Ouimette AP. Retention of Nitrate-N in Mineral Soil Organic Matter in Different Forest Age Classes. Ecosystems. 2019;22(6):1280-94.

Hamburg SP, Vadeboncoeur MA, Johnson CE, Sanderman J. Losses of mineral soil carbon largely offset biomass accumulation 15years after whole-tree harvest in a northern hardwood forest. Biogeochemistry. 2019;144(1):1-14.

Hooker TD, Compton JE. Forest ecosystem carbon and nitrogen accumulation during the first century after agricultural abandonment. Ecological Applications. 2003;13(2):299-313.

Hoover CM. Management Impacts on Forest Floor and Soil Organic Carbon in Northern Temperate Forests of the US. Carbon Balance and Management. 2011;6.

Ignace DD, Fassler A, Bellemare J. Decline of a foundation tree species due to invasive insects will trigger net release of soil organic carbon. Ecosphere. 2018;9(8).

Liu MY, Ussiri DAN, Lal R. Soil Organic Carbon and Nitrogen Fractions under Different Land Uses and Tillage Practices. Communications in Soil Science and Plant Analysis. 2016;47(12):1528-41.

Norris MD. Land-Cover Change in Western New York: Implications for Soil Carbon Dynamics. Northeastern Naturalist. 2012;19:89-100.

Orefice J, Smith RG, Carroll J, Asbjornsen H, Kelting D. Soil and understory plant dynamics during conversion of forest to silvopasture, open pasture, and woodlot. Agroforestry Systems. 2017;91(4):729-39.

Pacaldo RS, Volk TA, Briggs RD. No significant differences in soil organic carbon contents along a chronosequence of shrub willow biomass crop fields. Biomass & Bioenergy. 2013;58:136-42.

Paolucci AJ, Stolt MH. Assessing Dynamic Soil Properties in Southern New England Forests within an Ecological Site Framework. Soil Science Society of America Journal. 2018;82(5):1191-202.

Peach ME, Ogden LA, Mora EA, Friedland AJ. Building houses and managing lawns could limit yard soil carbon for centuries. Carbon Balance and Management. 2019;14(1).

Petrenko CL, Friedland AJ. Mineral soil carbon pool responses to forest clearing in Northeastern hardwood forests. Global Change Biology Bioenergy. 2015;7(6):1283-93.

Piche N, Kelting DL. Recovery of soil productivity with forest succession on abandoned agricultural land. Restoration Ecology. 2015;23(5):645-54.

Puhlick JJ, Fernandez IJ. Influence of mechanized timber harvesting on soil compaction in northern hardwood forests. Soil Science Society of America Journal. 2020;84(5):1737-50.

Puhlick JJ, Fernandez IJ. Change in soil carbon, nitrogen, and phosphorous after timber harvesting in northern hardwood forests. Soil Sci Soc Am J. 2023.

Puhlick JJ, Fernandez IJ, Weiskittel AR. Evaluation of forest management effects on the mineral soil carbon pool of a lowland, mixed-species forest in Maine, USA. Canadian Journal of Soil Science. 2016;96(2):207-18.

Puhlick JJ, Fraver S, Fernandez IJ, Weiskittel AR, Kenefic LS, Kolka RK, et al. Factors influencing organic-horizon carbon pools in mixed-species stands of central Maine, USA. Forest Ecology and Management. 2016;364:90-100.

Rao P, Hutyra LR, Raciti SM, Finzi AC. Field and remotely sensed measures of soil and vegetation carbon and nitrogen across an urbanization gradient in the Boston metropolitan area. Urban Ecosystems. 2013;16(3):593-616.

Richardson JB, Petrenko CL, Friedland AJ. Base cations and micronutrients in forest soils along three clear-cut chronosequences in the northeastern United States. Nutrient Cycling in Agroecosystems. 2017;109(2):161-79.

Richardson M, Stolt M. Measuring Soil Organic Carbon Sequestration in Aggrading Temperate Forests. Soil Science Society of America Journal. 2013;77(6):2164-72.

Richmond T, Germain RH, Brown K, Briggs R, Stehman S. Comparing single-wheel attachable tracks to chains and bare tires on skidders for mitigating soil disturbance during harvesting operations. International Journal of Forest Engineering. 2022.

Ross DS, Knowles ME. Partial harvest effects on the forest floor at four northern hardwood sites in the Green Mountains of Vermont USA. Forest Science. 2023; doi: 10.1093/forsci/fxad032.

Smith CT, Briggs RD, Stupak I, Preece C, Rezai-Stevens A, Barusco B, et al. Effects of whole-tree and stem-only clearcutting on forest floor and soil carbon and nutrients in a balsam fir (Abies balsamea (L.) Mill.) and red spruce (Picea rubens Sarg.) dominated ecosystem. Forest Ecology and Management. 2022;519.

Smith FC, Johnson AH, Dranoff M, Wibiralske A. Biomass and nutrient accumulation during natural afforestation of iron-smelting slag. Restoration Ecology. 1997;5(1):56-65.

Stolt MH, Drohan PJ, Richardson MJ. Insights and Approaches for Mapping Soil Organic Carbon as a Dynamic Soil Property. Soil Science Society of America Journal. 2010;74(5):1685-9.

Trammell TLE, Pataki DE, Pouyat RV, Groffman PM, Rosier C, Bettez N, et al. Urban soil carbon and nitrogen converge at a continental scale. Ecological Monographs. 2020;90(2).

Vario CL, Neurath RA, Friedland AJ. Response of Mineral Soil Carbon to Clear-Cutting in a Northern Hardwood Forest. Soil Science Society of America Journal. 2014;78(1):309-18.

Wang FM, Zhu WX, Chen H. Changes of soil C stocks and stability after 70-year afforestation in the Northeast USA. Plant and Soil. 2016;401(1-2):319-29.

Warren KL, Ashton MS. Change in Soil and Forest Floor Carbon after Shelterwood Harvests in a New England Oak-Hardwood Forest, USA. International Journal of Forestry Research. 2014;2014:527236.

Yanai RD, Arthur MA, Siccama TG, Federer CA. Challenges of measuring forest floor organic matter dynamics: Repeated measures from a chronosequence. Forest Ecology and Management. 2000;138(1-3):273-83.

Yavitt JB, Pipes GT, Olmos EC, Zhang JB, Shapleigh JP. Soil Organic Matter, Soil Structure, and Bacterial Community Structure in a Post-Agricultural Landscape. Frontiers in Earth Science. 2021;9.
